# Pharmaceutical Care Service at Primary Health Care Centers: An Insight on Patient Satisfaction

**DOI:** 10.1155/2022/6170062

**Published:** 2022-03-31

**Authors:** Manal Al Zaidan, Azza Mustafa Mohammed, Mohamed Izham Mohamed Ibrahim, Mashael Al Mahmoud, Samya Al Abdulla, Mohamed Ghaith Al-Kuwari

**Affiliations:** ^1^Director of Pharmacy, Primary Health Care Corporation, Doha, Qatar; ^2^Directorate of Strategy Planning and Health Intelligence, Primary Health Care Corporation, Doha, Qatar; ^3^Department of Clinical Pharmacy and Practice, College of Pharmacy, QU Health, Qatar University, Doha, Qatar; ^4^Clinical Affairs Directorate, Primary Health Care Corporation, Doha, Qatar; ^5^Directorate of Operations, Primary Health Care Corporation, Doha, Qatar

## Abstract

**Background:**

Patient's health care experiences and satisfaction are frequently used as a healthcare quality indicator.

**Aim:**

The study aims to evaluate the level of patient satisfaction with the pharmacy services provided at the Primary Health Care Corporation's (PHCC) pharmacies in Qatar.

**Methods:**

This study is a cross-sectional survey conducted in December of 2019. The study's setting is the Primary Health Care centers' pharmacies. All adult patients (≥18 years old) with mobile phone numbers documented on file who had their prescription orders filled at the PHCC's pharmacy units in 2019 were included in the study. Descriptive and inferential statistical methods were used to present the findings. The significance level was set at the alpha level of 0.05.

**Results:**

The usable responses were 9,564 from the total participants. Around 55.2% (*N* = 5,283) were males, 56.5% (*N* = 5,405) were in the age group (25–40), 19.2% (*N* = 1,837) were Qatari nationals, 39.7% (*N* = 3,801) had their medication dispensed in the Central Region of the State of Qatar, and 72.8% (*N* = 6,964) had at least undergraduate or higher degrees. The overall mean (SD) satisfaction score was (3.24 ± 0.629). Participants were less satisfied with their pharmacist's communication, especially offering information about medication's side effects (2.61 ± 1.2) and general health counselling (2.39 ± 1.2). Respondents were also less satisfied with waiting time (3.02 ± 1.3). Waiting time, gender, age, nationality, geographical region of the pharmacy, educational level, and familiarity with the PHCC were significantly associated with satisfaction level.

**Conclusion:**

The patients were reasonably satisfied, and the satisfaction level differed among different sociodemographic groups. Based on the public's needs and expectations, pharmacists need to continuously improve their effort to enhance the healthcare quality in the organization.

## 1. Introduction

Over the last number of decades, Qatar has come a long way in advancing its healthcare, establishing an accessible and efficient system, and strengthening the role of pharmacies in supporting the patients, especially at the primary care level [1, 2]. Qatar's National Development Strategy published in 2011 [2] supported the improvement of patients access to community pharmacies as well as advancing its efficiency through the evolution of the pharmacist role to become an important player in delivering core health services [1, 2]. As a result, primary care pharmacies have expanded their scope of pharmaceutical services (examples: assessing patient's medication treatments, by gathering information from the patient, caregiver(s), and/or medical record (CERNER); medication reconciliation to resolve any drug-related problems; interpreting lab values (i.e., warfarin); providing patient education and counselling; and reporting medication errors and adverse drug reactions). Pharmacists at the primary care setting can review and manage patients' drug therapy, assess drug-related needs, ensure medication and patient safety, and enhance medication adherence. In addition, they have moved from their traditional role of just selling and dispensing medications to embracing a patient-centered pharmacy model [3], utilizing effective communication and patient engagement [2, 3].

The Primary Health Care Corporation (PHCC) is the leading provider of primary health services in Qatar, and it operates through 27 health centers across all regions of the country. According to the PHCC annual statistical report, the PHCC has 2.6 pharmacists per 10,000 population, and in 2019, its health centers reported 2.4 million pharmacy visits [4]. The PHCC provides a wide range of pharmaceutical care services (as mentioned above) guided by evidence-based policies and procedures and supported by a multidisciplinary team of clinical staff and other external care providers [5]. PHCC's policies and procedures are compliant with Accreditation Canada International (ACI) standards and Qatar's Law. Examples of policies are related to dispensing of medications, prescribing of medications, medication reconciliation, and high-risk medications. Pharmacists are expected to educate the patients [6] about proper medication use, including drug dosage, storage, possible side effects, and drug interactions based on these policies and procedures. Moreover, they offer other public health services such as health promotion, lifestyle advice, medication optimization, and vaccinations [5].

In order to sustain the shift in the traditional role of the pharmacist, improve patients' outcomes, and optimize medication use, it is crucial to assess patients' satisfaction with the quality of care delivered to them through primary care pharmacies [7]. Gathering feedback from patients regarding their individual health care experiences and measuring their satisfaction through satisfaction surveys is frequently used as a healthcare quality indicator [8]. It is an important step towards involving patients in decision making and asserting their role as partners in advancing health services and their therapeutic outcomes [9]. Research shows that patients with a higher level of satisfaction are more likely to adhere to their medications and stay with the same medical care provider [10]. In addition, recognizing and tracking changes in patients' needs will allow stakeholders to identify potential areas of improvement and close the gap between patients' expectations and their actual experiences [8–10].

Some studies examined patients' satisfaction with Qatar's quality of pharmacy services [1, 11–13]. El hajj et al. (2011) reported that the public has a poor understanding of the community pharmacist's role in monitoring drug therapy, performing health screening, and providing drug information. Khudair and Raza (2013) indicated that patient satisfaction is positively influenced by service promptness, pharmacist attitude, medication counselling, pharmacy location, and waiting area. The study by Ghasoub et al. (2017) showed a high level of patients' satisfaction with the pharmacy layout and with the pharmacy staff [13]. However, limited studies evaluate patients' satisfaction at the primary care level. Therefore, our study's main objective is to assess the level of patient satisfaction with the pharmacy services provided at the Primary Health Care Corporation's (PHCC) pharmacies and its variation across different sociodemographic variables. We expect this study to fill the gap in the literature and provide current and relevant data to help maintain the continued efforts for quality improvement in Qatar's primary health care pharmaceutical services.

## 2. Methods

### 2.1. Study Design and Setting

This is an observational, cross-sectional survey study conducted from December 5^th^ to December 24^th^ of 2019 at the Primary Health Care Corporation's (PHCC) pharmacies. The present study was exempt from IRB approval (Ref no: PHCC/DCR/2021/07/051). Data were extracted as part of daily operational activities and were provided to researchers anonymized without personally identifiable information.

### 2.2. Survey Instrument

A self-administered, validated, and anonymous questionnaire was used. The questionnaire was adopted from another study conducted at Hamad Medical Corporation (HMC) in Qatar [13], and the questions were modified to suit the PHCC. The survey was piloted on 15 patients and administered in English and Arabic. The survey consisted of 12 questions divided into five sections. The first section consisted of five questions covering sociodemographic characteristics. The second section contained two questions that assessed familiarity with PHCC pharmacies. The third section looked at patient's satisfaction with the pharmacist in four statements. The fourth section had three questions that evaluated patients' satisfaction with pharmacy staff, waiting time, pharmacy location, design, and layout. Finally, the last section included one qualitative question asking about participants' further comments. The satisfaction data were collected using a five-point Likert scale. On the scale, the item “strongly unsatisfied” was given a score of “1,” and the items “unsatisfied,” “neutral,” “satisfied,” and “strongly satisfied” were given scores of “2,” “3,” “4,” and “5,” respectively.

The survey was developed on the web-based platform SurveyMonkey, and the links were sent via SMS (text messages).

The primary outcomes were as follows:

1. The overall mean satisfaction score of study participants with the services provided at PHCC's pharmacies;
2. The proportions of satisfaction scores of the patients with the pharmacist and information sharing;
3. The proportions of satisfaction scores of the patients with waiting times; and
4. The proportions of satisfaction scores of the patients with pharmacy location, design, and layout.

### 2.3. Study Population

No sampling was conducted. All the population was targeted with the online survey. All adult patients (≥18 years old) with mobile phone numbers documented on file who had their prescription orders filled at the PHCC's pharmacy units between January and December of 2019 and were willing to participate were included in the study. Participants who did not fit the age cohort criteria or could not read Arabic or English were excluded from the study.

### 2.4. Sample Size

Assuming an approximately normal sampling distribution, the minimum sample size required for the study to be executed was 665 for 99% confidence interval. The sample size was calculated using the formula:

(1) $$\begin{array}{c} n=\frac{\left(z^{2}P\left(1-P\right)/e^{2}\right)}{1+\left(z^{2}P\left(1-P\right)/e^{2}N\right)}, \end{array}$$

where *n* is the required sample size, *N* is the population size (504,744), *P* is the population proportions (set at 0.5), *z* is the value that specified the 99% confidence interval (= 2.58), *e* is the desired accuracy of sample proportions (set at ± 0.05).

### 2.5. Data Analysis

The data collected were analyzed using STATA/MP 15.1. Sociodemographic variables and satisfaction levels were described using frequencies, proportions, and mean and standard deviation (SD). We used the mean score to determine participants' satisfaction levels. Independent *t*-test and one-way ANOVA were used to evaluate the difference in satisfaction across different participants' groups. Post hoc tests were used to examine the difference among specific subgroups. The chi-square test was used to test the difference of proportions. Participants' comments on the qualitative question were analyzed using thematic analysis. We only included complete questionnaires; any survey with incomplete information was excluded from the analysis. All statistical tests were two-tailed, and a *p* value of ≤0.05 was considered the cutoff level for statistical significance.

## 3. Results

### 3.1. Sociodemographic Characteristics of Study Participants

The survey was sent to all persons who utilized the PHCC pharmacies between January and December of 2019, i.e., a total of 504,744. The collection yielded 15,238 survey responses that were received during the survey collection period. Out of all surveys received, 9,564 responses were included in the final analysis, which surpassed the desired 99% confidence interval threshold. Incomplete surveys were excluded from the analysis. Out of the total participants, 55.2% (*N* = 5,283) were males and 44.8% (*N* = 4,281) were females. Most of the participants 56.5% (*N* = 5,405) were in the age group (25–39), 80.8% (*N* = 7,727) belonged to non-Qatari nationalities, and 39.7% (*N* = 3,801) resided in the Central Region of the State of Qatar and were registered in one of the health centers in that region. Regarding the educational level, 72.8% (*N* = 6964) had undergraduate or higher degrees (Table 1).

Table 2 demonstrates participants experience and their familiarity with PHCC pharmacies. Out of participating patients, 88.5% (*N* = 8462) made at least one visit to a PHCC pharmacy during 2019, and most of these patients, 63.4% (*N* = 5364), had less than five visits during 2019.

### 3.2. Satisfaction Level of Study Participants with the Services Provided at PHCC's Pharmacies

The study participants were asked to rate their satisfaction with pharmaceutical services at the PHCC (see Table 3). Their overall mean satisfaction score was (3.24 ± 0.629) out of a maximum score of 5. This level will be used in our study as the cutoff score against which satisfaction level will be compared. Regarding participants' satisfaction with their communication with the pharmacist, the highest mean satisfaction score was given to the parameter “the pharmacist explained how to take your medication” (4.11 ± 0.8). However, the three other parameters were all given a mean score of less than the overall mean satisfaction score of 3.24. The parameter that was rated the lowest was “the pharmacist asked about any changes to your state of health since your last visit” (2.39 ± 1.2) followed by “the pharmacist informed you of the side effects associated with your medication” (2.61 ± 1.2).

When asked to rate their satisfaction with pharmacy staff, waiting time, and pharmacy location, design, and layout, participants were more satisfied with pharmacy staff (3.71 ± 1.0) and location, design, and layout (3.71 ± 1.1) than they were with waiting time. The overall satisfaction with waiting time (3.02 ± 1.3) was less than the cutoff score of 3.24.

### 3.3. Difference in Satisfaction Level among Study Participants' Groups

Based on sociodemographic groups, we have examined the difference in the mean satisfaction level with pharmacy staff communication and waiting time among participants. We decided to look deeply into these variables as they had lower mean satisfaction scores (refer to Table 4).

Regarding overall satisfaction with waiting time, gender, age, region of residence, educational level, and familiarity with PHCC were statistically significant differences. Females were significantly (*p* = 0.023) less satisfied with waiting time (2.99 ± 1.27) when compared to males (3.05 ± 1.32). Participants who are ≥60 years of age (3.51 ± 1.19) and those in the age group 40 to 59 years (3.15 ± 1.27) had significantly (*p* ≤ 0.001) higher mean satisfaction levels compared to those in the younger age group (≤39 years old). In addition, participants accessing pharmacies in the Central Region of the country (2.83 ± 1.32, *p* ≤ 0.001), who have higher educational degrees (2.96 ± 1.29, *p* ≤ 0.001), and those who visited PHCC pharmacies more than five times in 2019 (2.87 ± 1.35, *p* ≤ 0.001) were less satisfied with waiting times at pharmacies (Table 4). Although Qataris had a slightly lower mean satisfaction score when compared to Non-Qatari nationals, the difference was not statistically significant (*p* = 0.885).

When we examined the overall patients' satisfaction with the level of communication, we noticed a similar trend. Females, Qataris, those in the younger age group of ≤39, patients accessing pharmacies in the Central region Qatar, and those with higher educational degrees were significantly less satisfied with pharmacist communication and information sharing (Table 4).

### 3.4. Suggestions for Improvement

**Figure 1:**
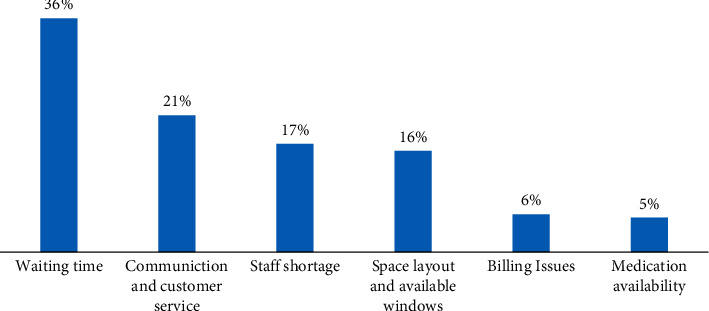
Participants' suggestions for improvement (*N* = 2,098).

The participants' comments and suggestions about what can be improved upon were analyzed, and the main themes identified are presented in Figure 1.

Most participants had suggestions on improving waiting time (36%, *N* = 751) and ways pharmacy staff communicate with patients (21%, *N* = 440). In addition, they have suggested changing the dispensing process and improving the queuing system to be more efficient for patients. This is consistent with the previous results as these factors had the lowest satisfaction scores. They have also proposed increasing the number of working staff each shift (17%, *N* = 348) and utilizing all the windows available for dispensing (16%, *N* = 326) to make the process more efficiently organized. Another suggestion was that billing should be conducted in the same window (6%, *N* = 122). This is because patients have to leave the dispensing window and pay at a different location within the health center, which can be inconvenient to customers. Finally, some patients (5%, *N* = 111) recommended improving medication availability.

## 4. Discussion

The present study evaluates patient satisfaction with the pharmacy services provided at the PHCC pharmacies in Qatar, and its variation across different sociodemographic variables. Understanding the public's needs and expectations is a key factor in improving PHCC's pharmacies' services. It is a big part of Qatar's continuous healthcare quality improvement efforts. The knowledge gained from patient satisfaction surveys can help health care providers, especially pharmacists, meet patients' needs and improve the quality of the services provided. In addition, evidence shows that greater patient satisfaction is associated with better medication compliance and enhanced persistence [8].

In the present study, patients were highly satisfied with the pharmacy staff and how the pharmacist explained the proper way of taking medications. However, patients were less satisfied with communication with their pharmacist, especially regarding offering information related to medication side effects and counselling them about their overall health. Our results were similar to a previous study conducted in Qatar by El Hajj et al. (2011) in which patients rated communication skills (100% of respondents), medication knowledge (98%), and understanding of patients' concerns (93%) as top pharmaceutical care qualities [11]. However, another study conducted in the UAE reported that only “25% to 30% of respondents agreed that the pharmacist explains all possible side effects, provides information on proper storage of medication and drug therapy and/or disease” [6]. On the other hand, a study by Ghasoub et al. (2017) reported that 94% of the participants screened for satisfaction in the outpatient pharmacies at Hamad Medical Corporation (HMC) specialized tertiary hospitals in Qatar were satisfied with medication information on clinical efficacy shared by pharmacy staff [13].

Participants were less satisfied with the waiting time to dispense medications. Research documented that there is a positive relationship between patient satisfaction and service promptness. For example, Khudair et al. (2013) studied factors associated with pharmaceutical service patients' satisfaction, and they found a statistically significant positive relationship between satisfaction and waiting time [12, 14, 15].

Satisfaction level was influenced by sociodemographic characteristics such as age, gender, nationality, region of residence, number of previous visits to pharmacies, and education level. For example, patients in the older age group (≥60) were more satisfied with the pharmacy services than the other age groups, which is consistent with findings from Alturki et al. (2013) and Ayele et al. (2020) [14, 15]. Moreover, females were generally less satisfied than males, and participants with a higher education level also had a lower satisfaction rate. This could be due to lower awareness and expectations about the service provided at pharmacies among patients with less education [15].

This study found that Qataris were less satisfied with the communication level at the pharmacies and the waiting time. However, the difference was not statistically significant for waiting time. This finding is consistent with Al-Kuwari et al. (2009), as they found that Qatari participants were less satisfied with primary healthcare services when compared to non-Qataris [12, 16]. Moreover, Khaled et al. [17] reported similar findings. They explained that the recent increase in national wealth and, by extension, the big increase in the wealth of the citizens might have created expectations of what “should be” and led to continuously high expectations for a high-quality standard from the Qataris toward the services in the public sector. Additionally, patients that accessed PHCC pharmacies in the Central region of Qatar were less satisfied than those in the Western and Northern regions. The Central region has a higher population density and includes the biggest cities in Qatar, namely, Doha and Al Wakra. Around two-fifth (531,568) of the PHCC's patients are from that region, resulting in busier health centers than other regions [4].

When asked to propose possible further improvements, participants offered many suggestions related to waiting time, better communication, and improved space layout. These suggestions are consistent with the previous study [18] on pharmaceutical services. For example, Kerr et al. suggested that the development of patient-pharmacist communication skills should start at the undergraduate level and continue through the whole career of the pharmacist, acknowledging the importance of that skill. The study sample was primarily educated at a bachelor's level or higher, explaining the expectation of better information communication. Pharmacy departments in the PHCC should review and improve the dispensing services and pharmacy workflow. In addition, the policymaker should review the policy concerning coverage and payment for medication. For example, there is a need for a clear process for getting the exemption of payment for a particular population category such as kids under Qatari mother with non-Qatari father.

### 4.1. Strengths and Limitations

A limited number of recent studies evaluate patients' satisfaction with pharmaceutical services at the primary care level. To measure patient satisfaction in our study, we used a newly developed survey questionnaire that specifically targeted patients attending primary health care centers. Moreover, the responses were from all 27 health centers covering almost all of Qatar's geographical regions. However, the study did present some limitations. We used nonrandom sampling methods as the survey was offered exclusively through links sent via text messages, introducing nonresponse bias, especially among older cohorts that tend to use the Internet less frequently than the younger ones [19]. In addition, clients who could not read Arabic or English were excluded from the study. Social desirability bias may also negatively affect the responses. The respondents might be over-reporting their specific satisfaction toward the pharmacists or overall satisfaction toward the pharmacy. All these limitations may not have represented the actual satisfaction level of the PHCC patients.

### 4.2. Study Recommendations

We recommend that the PHCC empower the pharmacists to move from the traditional practice of only dispensing medications to taking on a more proactive and counselling role in the care of their patients. This can be achieved through organizational change and enhanced training. Moreover, we recommend surveying patients for their satisfaction with pharmaceutical services annually, reporting the results as key performance indicators. It is essential to further research with more detailed and comprehensive questions about patients' experiences, health promotion, referral to family doctors, or other factors that might affect their satisfaction. Studies can also be carried out to survey pharmacists to learn about the barriers they face in providing pharmaceutical care, e.g., barriers hindering their ability to provide education beyond how patients should take their medications.

## 5. Conclusions

In summary, our findings demonstrate that the satisfaction level was reasonable and differed among different sociodemographic groups. Patients were more satisfied with the pharmacist's explanation of how to take their medication but less satisfied with the explanation of the side effects, how the medication works, and changes in the disease state. In addition, the patients were less satisfied with the waiting time. Understanding the public's needs and expectations is a key factor in continuous healthcare quality improvement efforts at the PHCC.

## 6. Impact of Findings on Practice

- Female patients, Qataris, those in the younger age group of 18–39, patients accessing pharmacies in the Central region Qatar, and those with higher educational degrees were less satisfied with pharmacist communication and information sharing.
- Patients were highly satisfied with the communication aspect: “the pharmacist explained how to take your medication.”
- Pharmacists should move from the traditional practice of only dispensing medications to taking on a more proactive and counselling role in the care of their patients.

## Figures and Tables

**Table 1:** Sociodemographic characteristics.

*Gender*	N	%
Female	4,281	44.8
Male	5,283	55.2

*Age group*		
>25	658	6.9
25–39	5,405	56.5
40–59	3,162	33.1
≥60	339	3.5

*Nationality*		
Qatari	1,837	19.2
Non-Qatari	7,727	80.8
Educational level		
Secondary school diploma	2,600	27.2
Undergraduate degree	4,575	47.8
Postgraduate degree	2,389	25.0

*Geographical region*		
Central	3,801	39.7
Northern	2,727	28.5
Western	3,036	31.7
Total	9,564	100%

**Table 2:** Participants familiarity with PHCC.

Familiarity with PHCC	N	%
Did you visit the health center pharmacies in 2019		
Yes	8462	88.5
No	1102	11.5
Total	9564	100%

Number of visits to health center pharmacies in 2019		
One visit	1339	15.8
(2–4) visits	4025	47.6
(5–10) visits	1694	20.0
More than 10	1404	16.6
Total	8462	100%

**Table 3:** The proportions and mean of satisfaction scores, *n* = (9564).

	Strongly unsatisfied *n* (%)	Unsatisfied *n* (%)	Neutral *n* (%)	Satisfied *n* (%)	Strongly satisfied *n* (%)	Mean (SD)
Patient's satisfaction with the pharmacist						
The pharmacist explained how to take your medication	185 (1.9)	254 (2.7)	906 (9.5)	5183 (54.2)	3036 (31.7)	4.11 (0.8)
The pharmacist explained how the medication works and its effect	1182 (12.4)	1772 (18.5)	2234 (23.4)	3226 (33.7)	1150 (12)	3.15 (1.2)
The pharmacist informed you of the side effects associated with your medication	2221 (23.2)	2572 (26.9)	2191 (22.9)	1866 (19.5)	714 (7.5)	2.61 (1.2)
The pharmacist asked about any changes to your state of health since your last visit	2647 (27.7)	2835 (29.6)	2259 (23.6)	1333 (13.9)	490 (5.1)	2.39 (1.2)

Patients' overall satisfaction						
Your overall satisfaction with pharmacy staff	377 (3.9)	747 (7.8)	1967 (20.6)	4646 (48.6)	1827 (19.1)	3.71 (1.0)
Your overall satisfaction with pharmacy location, design, and layout	521 (5.5)	801 (8.4)	1639 (17.1)	4588 (48)	2015 (21.1)	3.71 (1.1)
Your overall satisfaction with the waiting time	1701 (17.8)	1763 (18.4)	1780 (18.6)	3252 (34)	1068 (11.2)	3.02 (1.3)

**Table 4:** Difference in the mean satisfaction level by sociodemographic characteristics.

	Overall satisfaction with waiting time		Overall satisfaction with communication with pharmacy staff	
Variable	Mean (SD)	Significance level (2 sided)^*∗*^	Mean (SD)	Significance level (2 sided)

Gender		0.023		*P* ≤ 0.001^*∗∗*^
Female	2.99 (1.27)		2.98 (0.87)	
Male	3.05 (1.32)		3.12 (0.93)	

Nationality		0.885		*P* ≤ 0.001^*∗∗*^
Qatari	3.02 (1.29)		2.88 (0.92)	
Non-Qatari	3.03 (1.31)		3.11 (0.89)	

Age		*P* ≤ 0.001		*P* ≤ 0.001^*∗∗∗*^
<25	2.86 (1.33)		3.0 (0.97)	
25–39	2.94 (1.3)		3.05 (0.9)	
40–59	3.15 (1.27)		3.07 (0.89)	
≥60	3.51 (1.19)		3.27 (0.88)	

Region		*P* ≤ 0.001		*P* ≤ 0.001^*∗∗∗*^
Central	2.83 (1.32)		2.97 (0.9)	
Northern	3.19 (1.27)		3.15 (089)	
Western	3.12 (1.27)		3.1 (0.9)	

Educational level		*P* ≤ 0.001		*P* ≤ 0.001^*∗∗∗*^
Secondary school diploma	3.13 (1.31)		3.25 (0.91)	
Undergraduate degree	3.03 (1.28)		3 (0.87)	
Postgraduate degree	2.96 (1.29)		2.97 (0.92)	

Familiarity with PHCC (based on visits' number)		*P* ≤ 0.001		0.899^*∗∗*^
(1–4) visits	3.11 (1.25)		3.1 (0.88)	
>5 visits	2.87 (1.35)		3.1 (0.93)	

*∗*Chi-square test was used at alpha = 0.05; ^*∗∗*^Independent *t*-test was used at alpha = 0.05; ^*∗∗∗*^One-way ANOVA was used at alpha = 0.05.

## Data Availability

The data were extracted as part of daily operational activities and was provided to researchers anonymized without personally identifiable information.

## References

[1] Ibrahim M. I., Palaian S., Al-Sulaiti F., El-Shami S. (2016). Evaluating community pharmacy practice in Qatar using simulated patient method:acute gastroenteritis management. *Pharmacy Practice*.

[2] https://www.psa.gov.qa/en/knowledge/HomePagePublications/Qatar_NDS_reprint_complete_lowres_16May.pdf.

[3] Wolters M., van Hulten R., Blom L., Bouvy M. L. (2017). Exploring the concept of patient centred communication for the pharmacy practice. *International Journal of Clinical Pharmacy*.

[4] Primary Health Care Corporation (2019). *Annual Statistical Report: A Strategy Planning & Health Intelligence Directorate Initiative*.

[5] Primary Health Care Corporation (2020). Pharmaceutical care service.

[6] El-Sharif S., Alrahman N., Khaled N., Sayah N., Gamal E., Mohamed A. (2017). Assessment of patient’s satisfaction with pharmaceutical care services in community pharmacies in the United Arab Emirates. *Archives of Pharmacy Practice*.

[7] Surur A. S., Teni F. S., Girmay G., Moges E., Tesfa M., Abraha M. (2015). Satisfaction of clients with the services of an outpatient pharmacy at a university hospital in northwestern Ethiopia: a cross-sectional study. *BMC Health Services Research*.

[8] Ayalew M., Taye K., Asfaw D. (2017). Patients’/Clients’ expectation toward and satisfaction from pharmacy services. *Journal of Research in Pharmacy Practice*.

[9] Al-Abri R., Al-Balushi A. (2014). Patient satisfaction survey as a tool towards quality improvement. *Oman Medical Journal*.

[10] Asadi-Lari M., Tamburini M., Gray D. (2004). Patients’ needs, satisfaction, and health related quality of life: towards a comprehensive model. *Health and Quality of Life Outcomes*.

[11] El Hajj M., Mansoor S., El Salem S. (2011). Public’s attitudes towards community pharmacy in Qatar: a pilot study. *Patient Preference and Adherence*.

[12] Fahmi Khudair I., Asif Raza S. (2013). Measuring patients’ satisfaction with pharmaceutical services at a public hospital in Qatar. *International Journal of Health Care Quality Assurance*.

[13] R G., M Z., Yafie S A., K A. S., Y R., Ibrahim Mi M. (2017). Preliminary study of patients’ perceptions and satisfaction in outpatient pharmacies at the cancer and heart centres in Qatar. *Journal of Applied Pharmacy*.

[14] Alturki M., Khan T. M. (2013). A study investigating the level of satisfaction with the health services provided by the Pharmacist at ENT hospital, Eastern Region Alahsah, Kingdom of Saudi Arabia. *Saudi Pharmaceutical Journal*.

[15] Ayele Y., Hawulte B., Feto T., Basker G. V., Bacha Y. D. (2020). *Assessment of Patient Satisfaction with Pharmacy Service and Associated Factors in Public Hospitals, Eastern Ethiopia*.

[16] Al-Kuwari M., Emadi N., Falamarzi S., Ansari A. (2009). Patients’ satisfaction with primary health care services in Qatar. *Middle East Journal Of Family Medicine*.

[17] Khaled S. M., Shockley B., Abdul Rahim H. F. (2017). The effects of citizenship status on service utilization and general satisfaction with healthcare: a cross-cultural study. *Journal of the International Society for Quality in Health Care*.

[18] Kerr A., Strawbridge J., Kelleher C. (2017). How can pharmacists develop patient-pharmacist communication skills? A realist review protocol. *Systematic Reviews*.

[19] Ibrahim M. I., Palaian S., Al-Sulaiti F., El-Shami S. (2016). Evaluating community pharmacy practice in Qatar using simulated patient method:acute gastroenteritis management. *Pharmacy Practice*.

